# Astrocytes at the Hub of the Stress Response: Potential Modulation of Neurogenesis by miRNAs in Astrocyte-Derived Exosomes

**DOI:** 10.1155/2017/1719050

**Published:** 2017-09-07

**Authors:** Alejandro Luarte, Pablo Cisternas, Ariel Caviedes, Luis Federico Batiz, Carlos Lafourcade, Ursula Wyneken, Roberto Henzi

**Affiliations:** ^1^Centro de Investigaciones Biomédicas, Facultad de Medicina, Universidad de los Andes, Santiago, Chile; ^2^Biomedical Neuroscience Institute, Universidad de Chile, Santiago, Chile; ^3^Cells for Cells, Santiago, Chile

## Abstract

Repetitive stress negatively affects several brain functions and neuronal networks. Moreover, adult neurogenesis is consistently impaired in chronic stress models and in associated human diseases such as unipolar depression and bipolar disorder, while it is restored by effective antidepressant treatments. The adult neurogenic niche contains neural progenitor cells in addition to amplifying progenitors, neuroblasts, immature and mature neurons, pericytes, astrocytes, and microglial cells. Because of their particular and crucial position, with their end feet enwrapping endothelial cells and their close communication with the cells of the niche, astrocytes might constitute a nodal point to bridge or transduce systemic stress signals from peripheral blood, such as glucocorticoids, to the cells involved in the neurogenic process. It has been proposed that communication between astrocytes and niche cells depends on direct cell-cell contacts and soluble mediators. In addition, new evidence suggests that this communication might be mediated by extracellular vesicles such as exosomes, and in particular, by their miRNA cargo. Here, we address some of the latest findings regarding the impact of stress in the biology of the neurogenic niche, and postulate how astrocytic exosomes (and miRNAs) may play a fundamental role in such phenomenon.

## 1. The Relevance of the Hippocampus in the Stress Response

Stressful life events are strong precipitating factors of neuropsychiatric pathologies including mood disorders such as major depression (MD) or bipolar disorder (BD) [1]. Stress can be defined as any adaptive mechanism triggered to recover the organism's homeostasis, composed of a vast array of modifications in the physiology of different organs, including the central nervous system (CNS) at different scales, that is, plastic changes which range from molecular dynamics to behavioral adaptations [2].

The proper adaptive response to stressors is known as “stress resilience” and the multiple biological processes underlying resilience are collectively termed allostasis [3]. Nevertheless, plastic changes can be deleterious to cerebral and overall body health under prolonged stress (reviewed in [4]). Furthermore, increasing evidence shows that stress impacts the induction not only of psychiatric but also systemic pathologies such as cardiovascular diseases, cancer, and inflammation-related diseases [5–7].

The mechanisms that participate in the stress response involve the CNS, where the hypothalamus-pituitary-adrenal axis (HPA) has a central role. HPA activation leads to an increase in the systemic levels of glucocorticoids (GCs) (cortisol in humans and corticosterone in rodents) in concomitance with changes in the activity of the autonomic system, with norepinephrine and epinephrine as final products [2]. GCs are key hormones of the stress response that are able to cross the blood-brain barrier due to their lipophilic nature. Receptors in target cells include the high-affinity mineralocorticoid receptor [8] or the low-affinity glucocorticoid receptors [9]. In the brain, both receptors are mainly occupied by GCs and translocate to the nucleus after binding to their ligand, where they modify the expression of different genes that govern the stress response. The brain area profoundly affected during chronic stress is the hippocampus. The human and rodent hippocampi correspond to a CNS region where glucocorticoid receptors (GRs) are expressed in neurons, astrocytes as well as in some neural stem cells [10–12], conferring a high sensitivity of this forebrain structure to changes in glucocorticoids levels [13]. A negative feedback loop mediated by cortisol regulates the activity of the HPA by targeting structures such as the paraventricular nucleus and the hippocampus. In the latter, synaptic inputs can directly exert an overall inhibitory effect on the activity of the HPA [14]. Stress triggers molecular and structural changes in the hippocampus, including dendritic and spine atrophy that is concomitant to downregulation of specific synaptic protein [15, 16]. Many of these glucocorticoid-mediated changes can be mimicked by exogenous application of these corticosteroids (extensively reviewed by [13]).

Intriguingly, the hippocampus harbors one of the two identified brain structures in mammals that retains the capacity to generate new neurons in adulthood, that is, the neurogenic niche of the subgranular zone (SGZ) in the dentate gyrus (DG). The process by which new neurons are continuously generated in the SGZ of adults is known as adult neurogenesis and implies the self-renewal, proliferation/activation of neural stem/precursor cells, their differentiation into neurons, as well as their migration, maturation, and even their integration into the hippocampal functional circuits [17–19]. Any modification in one of these stages can influence (positively or negatively) the generation of new neurons, and diverse pathological conditions including chronic stress have been described to decrease adult hippocampal neurogenesis [20]. Conversely, antidepressant interventions show an increase in the number of neural stem/precursor cells in the DG. In fact, some antidepressant drugs depend on neurogenesis to induce recovery from depressive symptoms [21–24].

Hippocampal newborn neurons are essential for the proper endocrine and behavioral adaptation to stress [25], and SGZ neurogenesis contributes to the negative feedback on the HPA axis, as its disruption induces a larger response to a mild stressor [26]. Consistently, it has been described that altered neurogenesis leads to a slower recovery of GC levels after stress [27], suggesting a cross talk between hippocampal neurogenesis and the HPA axis. Likewise, reduced neurogenesis is associated with impaired responsiveness of the HPA axis in the dexamethasone suppression test [28]. Thus, any process that restores hippocampal neurogenic activity might contribute to better cope with stress. This could take place at the various stages involved in this process, from cell proliferation to the generation of mature DG neurons.

## 2. The Adult Hippocampal Neural Stem/Precursor Cells

Seri and coworkers [29] observed for the first time that neural stem cells that undergo proliferation in the SGZ display radial glia characteristics expressing the glial fibrillary acidic protein (GFAP), in addition to markers of undifferentiated cells such as vimentin, SOX2, and Nestin. SGZ stem cells are called type 1 cells (reviewed by Ming and Song [30]). These give rise, through asymmetric division, to highly proliferative intermediate progenitors known as type 2a (positive for Nestin and PSA-NCAM and negative for GFAP) and 2b cells (positive for Tbr2 and PSA-NCAM). The latter cells give rise to neuroblasts or type 3 cells (positive for doublecortin, PSA-NCAM, and NeuN) that migrate into the inner granular layer. Within days, type 3 cells will become immature neurons that, after about 4 weeks, extend dendrites towards the molecular layer and project axons through the hilus toward the CA3 (reviewed by Zhao et al., Covic et al., and Bonaguidi et al. [17, 31, 32]). In summary, both neural stem and progenitor cells coexist in the SGZ and can generate new granule neurons [33, 34]. In the present review, we will use the acronym NSPCs to describe both neural stem cells and precursor cells.

## 3. Magnitude of Adult Hippocampal Neurogenesis

It has been estimated that in the rat hippocampus 9000 new cells are generated every 25 hours [35]. In mice, on the other hand, the number is much lower: only 2700 new cells per day are generated [36]. After 30 days, ~30% of new cells survive and differentiate into mature neurons with complex dendritic and axonal structure. In humans, direct evidence of adult neurogenesis has been provided first by the use of the synthetic analog BrdU (bromodeoxyuridine, 5-bromo-2′-deoxyuridine) [37] and later on by an elegant publication which presented an integrated model of cell turnover dynamics in the hippocampus by measuring the concentration of nuclear bomb test-derived ^14^C in hippocampal cells. This work shows that one-third of human hippocampal neurons are exchanged throughout life and that 700 new neurons are added per day. The authors calculated a turnover of 1.75% newborn neurons per year that decreased modestly during aging. Taken together, this data indicates that adult hippocampal neurogenesis is not a minor process and may contribute significantly to human brain function during physiology and disease [38].

## 4. Adult-Born Hippocampal Neurons and the Impact of Stress

After stressful experiences, the activation of the HPA axis and the elevation of systemic GC levels lead to the impairment of NSPC proliferation in the SGZ both in developmental stages as well as in adulthood [39, 40].

Consistently, adrenalectomy increases the formation of new neurons in young and aged rodents [41–43]. Furthermore, the hyporesponsive stress period in rats (from 2 days after birth to 2 weeks old), characterized by low basal levels of GCs and a diminished response to stress [44], is associated with the maximal neurogenesis period in the SGZ [45, 46]. Likewise, adrenalectomy prevents the suppression of neurogenesis induced by stress [47, 48]. The effect of GCs on the neurogenic potential has shown to be dose-dependent in a human hippocampal progenitor cell line. Interestingly, low concentrations of cortisol stimulate proliferation and gliogenesis and decrease neurogenesis by signaling through mineralocorticoid receptors. On the other hand, high doses of cortisol decreased proliferation through glucocorticoid receptor signaling, with no effect on gliogenesis [49]. Similarly, decreased neurogenesis has been observed in different stress models, including chronic and acute stress, for example, subordination stress [50], resident-intruder stress [51], footshock [52], restraint stress [53, 54], or stress-induced by isolation [55] and predator odor [56]. It is worth remarking that stress has been shown to affect neurogenesis in a reduced window of time. Tanapat et al. observed that animals may experience a rebound in cell proliferation after the initial stress-induced suppression to compensate the alteration [47]. These results agree with several publications in which acute stressful experiences increases neurogenesis [57–60].

Despite significant advances in the field of neurogenesis and stress over the past two decades, detailed mechanisms underlying the inhibition of cell proliferation under stress conditions and its adaptations remain unknown.

## 5. Astrocytes Are Key Players in Adult Neurogenic Niche

Neurogenesis is regulated through its specialized microenvironment, the neurogenic niche. In adult mammals, including humans, neurogenic niches are concentrated in restricted areas; the most commonly described are the subventricular zone (SVZ) of the lateral ventricles and, mentioned above, the SGZ of hippocampal DG [61, 62]. The regulation of neurogenesis in the neurogenic niche is such that NSPCs obtained from exogenous SVZ and grafted into another SVZ host are able to generate new neurons; but NSPCs from the SVZ grafted into nonneurogenic brain regions show a scarce neurogenic potential, suggesting that here, a very particular cellular and molecular context accounts for the control of neurogenesis [63, 64].

Any cellular type within the niche can influence the neurogenic process by diffusible signals or by cell-cell interactions. In the SGZ, the main cellular components are astrocytes, endothelial cells, pericytes, oligodendrocytes, microglia, different types of neurons present in the DG, and the aforementioned NSPCs [65]. Although each cell type may have a significant contribution to the neurogenic process, in the present review, we will focus on the role of astrocytes as key elements in the control of the neurogenic process under stress.

Astrocytes subserve a myriad of functions that have been described both *in vitro* and *in vivo* (extensively reviewed by Khakh and Sofroniew [66]). In the hippocampus, protoplasmic astrocytes extend their processes radially and some of them contact blood vessels to form perivascular end feet of the blood-brain barrier (BBB), while others may contact neurons (e.g., tripartite synapse) or be coupled to oligodendrocytes through connexins [67]. In addition, astrocytes may connect with other astrocytes through connexins, generating a sort of functional syncytium able to signal by propagating calcium waves along several distant cells *in vivo* [68]. It is therefore not surprising that they are thought to have a central role in the functional output of the neurogenic process [69]. For example, astrocytes negatively influence the differentiation of NSPCs after the activation of jagged1-mediated Notch pathway by cell-cell contact [70] or by the secretion of growth factors such as insulin-like growth factor binding protein 6 (IGFBP6) and decorin [71]. On the contrary, released factors such as Wnt3a, neurogenesin-1, and different interleukins such as IL-1*β* and IL-6 or cell-to-cell contact mediated by ephirn-B2 signaling positively regulate neurogenesis [29, 71–74]. Thus, it is possible that, depending on the physiological and anatomical context, the astrocyte secretome has distinct effects on the neurogenic process [71]. In this line, hippocampal astrocytes are more efficient than cortical astrocytes in promoting neuronal differentiation of NSPCs [75].

The secretory activity of astrocytes in the DG mediates the synaptic and network integration of newborn neurons *in vivo*, highlighting their role as key mediators of the functional output of neurogenesis [76]. Previous data supports this view, as astrocytes promote the differentiation of progenitor cells and control the maturation and synaptic integration of newborn neurons *in vitro* [77, 78].

## 6. Stress, Astrocyte Plasticity, and Neurogenesis

A wide body of evidence has shown that acute and/or chronic stress can alter the morphology and functionality of different glial cell types in the brain, such as microglia [79, 80], oligodendroglia [81], and astrocytes [82].

Czéh et al. observe that tree shrews subjected to 5 weeks of psychosocial stress showed a 25% reduction in the intermediate filament protein of astrocytes GFAP, as well as a 25% reduction in the somatic volume of hippocampal astrocytes [83]. In the past few years, several publications using other stress protocols have led to similar observations [84–86]. Nevertheless, some publications using the chronic restraint model have reported an increase in GFAP positive cell number and in the protein level in the hippocampus [87, 88].

Other proteins expressed by astrocytes such as connexin 30 and 43 (gap junction proteins), the water channel aquaporin-4 (AQP4), the calcium-binding protein S100*β* and the amino acid transporters 1 and 2 (EAAT1, EAAT2), and glutamine synthetase have altered expression levels in both animals models of stress and in human brain samples analyzed postmortem compared with controls (reviewed in [89]). Despite the importance of some of these proteins in calcium homeostasis, there is a lack of studies showing how astrocytic calcium metabolism is regulated under stress conditions.

Moreover, a recent publication by Zhao et al. has shown that a decrease in glycogen content is associated with chronic stress, being one of the main mechanism in astrocytes capable of inducing their structural and molecular alterations. This result may be of importance as it moves away from the GC-centered theory of stress [90].

On the other hand, different publications have reported that when astrocytes are exposed to high levels of GCs, GC bound to GRs translocates to the nucleus and enhances the expression of genes related with neurogenesis, one example is the Fgf2 gene [91]. FGF2, the protein encoded by Fgf2, is a potent and necessary proliferative factor in adult NSPCs [92]. Nevertheless, other different effects mediated by astrocytes over the adult neurogenesis after a stressful condition have not been fully unveiled. In Table 1, we resume the main effects described for this issue, both *in vivo* and *in vitro*.

## 7. Exosomes Biogenesis and the Relevance of Their Content in Controlling Cellular Function

In addition to soluble components (see Section 5), the astrocyte secretome contains extracellular vesicles (EVs) such as exosomes [93] that represent a different source of cell-cell communication [94, 95]. Exosomes are generated in the endocytic pathway after the invagination and subsequent fission of a domain in the endosomal membrane that give rise to an exosome precursor called intraluminal vesicle (ILV) of the multivesicular body (MVB). After the fusion with the plasma membrane, the ILVs are released into the extracellular space as spherical vesicles of 40–100 nm, called exosomes [96]. The biogenesis of exosomes requires different molecular components including the mechanisms dependent of the ESCRT (endosomal sorting complex required for transport) machinery [97, 98] and lipid-dependent mechanisms [99, 100]. Proteins that participate in their biogenesis are frequently used as positive markers of exosomes, as well as proteins associated with lipid rafts and tetraspanins such as Alix, flotillin, TSG101, and CD63 [101].

Exosomes contain a complex molecular cargo that include proteins, lipids, and nucleic acids that may be biologically active on recipient cells [102]. The protein composition is diverse and depends on the cellular type and the physiological context; nevertheless, as they originate in the endocytic pathway, the most common proteins independent of the cell type of origin are related to vesicular transport and fusion (Rab GTPasas, SNAREs, annexins, and flotillin), different integrins and tetraspanins (CD63, CD9, CD81, and CD82), and heat shock proteins (Hsc/Hsp 70 and 90) and proteins implicated in the biogenesis of MVB (Alix and TSG 101) [103]. Regarding their lipidic content, one characteristic of the exosomes is their enrichment in lipid rafts including cholesterol, sphingolipids (such as ceramide), and glycerophospholipids with long and saturated fatty acyl chains [101]. Finally, among the most relevant biologically active molecules present in exosomes are nucleic acids, particularly small noncoding RNAs such as miRNAs (see below).

Exosomes play a significant role in the secretome of a given cell, subserving functions in the communication between cells [104]. Furthermore, virtually all eukaryotic cells release exosomes and are capable of taking them up [105, 106]. Regarding the CNS, oligodendrocytes, neurons, astrocytes, and microglia are capable of releasing exosomes with functional consequence on neuronal physiology [107]. Actually, exosomes have been proposed to be key players in the pathogenesis of different CNS diseases, including neurodegenerative diseases, infectious diseases, neuroinflammation, and even psychiatric disorders such as depression [108, 109]. Considering the high molecular diversity and complexity of their cargo, a fundamental question to understand the biological relevance of astrocytic exosomes in neurogenesis is a critical analysis of the relevant molecular cargo that could potentially control the fate of NSPCs and the neurogenic process.

So far, the functional transfer/interaction of exosomes to target cells has been shown mostly *in vitro*, but there is increasing data being obtained *in vivo*. Analysis of *in vivo* evidence is crucial as it settles the basis to propose that astrocytes within the neurogenic niche might be able to modify NSPCs' physiology through functional transfer of exosomal cargo in physiological conditions and during diseases. In this regard, an outstanding result came from the work of Zhang et al. where they found *in vivo* that tumor cells lose the expression of the tumor suppressor protein phosphatase and tensin homolog (PTEN) after incorporating astrocytic exosomes, due to the presence of a microRNA (miRNA) that targets PTEN [110].

Thus, although still speculative, we discuss a putative scenario where astrocytes in the neurogenic niche modulate the cellular behavior of NSPCs on the virtue of exosome transfer. It is important to notice that, in the literature, several of the functional effects described for exosomes are attributed to mRNA or miRNA transfer rather than proteins or lipids (as an example see [111]), though there is a growing interest to examine the relevance of these molecules in the exosomal cargo.

## 8. miRNAs in Astrocyte-Derived Exosomes as Modulators of Adult Neurogenesis and Stress Response

miRNAs are small noncoding RNAs (20–22 nucleotides) that cause deadenylation as well as translational repression of mRNAs by binding to their 3′ untranslated region (3′UTR). They have been proposed to be integral regulatory molecules in both physiological conditions and in disease states, because a single miRNA molecule can repress several hundreds (and even thousands) of mRNA molecules [112, 113]. Furthermore, the targeting of a single mRNA by a miRNA can potentially modulate the transcription of a vast array of proteins [114].

miRNAs are known to be a key element for neuronal differentiation; for example, Kawase-Koga et al. observed that NSPCs undergo cell death and affecting also the neuronal differentiation and their maturation after conditionally deleting the expression of the RNAse III enzyme DICER, an enzyme that processed miRNA precursor into mature miRNAs in specific stages of mice development [115]. Another miRNA that has also proved to modulate neuronal differentiation is miR-124, which contributes to the downregulation of Ezh2, a histone H3 Lys-27 histone methyltransferase that governs the transcription of several neuron-specific genes, diminishing the differentiation of mouse embryonic NSPCs as a final outcome [116, 117].

On the other hand, an increase in the expression of miR-9 in neurogenic regions leads to a reduction of NSPC proliferation and accelerated neural differentiation due to its modulation of TLX, a key regulator of NSPCs self-renewal, whereas the knock-in of miR-9 leads to increased proliferation of NSPCs [118]. Other miRNAs such as miR-128 and miR-137 promote differentiation of NSPCs, while their knockdown compromises their self-renewal [119].

Recently, Han et al. have shown that miRNA-19 (a member of polycistronic miRNA genes critical for brain development) is enriched in NSPCs and decreases during neuronal development. They found that this miRNA controls the maturation and positioning of newborn neurons in the granular cell layer of the DG by suppressing Rap guanine nucleotide exchange factor 2 (Rapgef2) [120]. In another study, the authors found that miR-20 downregulates the transcriptional repressor gene REST, inhibiting the differentiation of NSPCs [121]. Other miRNAs controlling both proliferation and differentiation of adult NSPCs are miR-137 [122] and rno-miR-592 [123]. Taken together, these data indicate an important participation of miRNAs in adult neurogenesis.

Multiple evidence has shown a relationship between miRNAs and stress, both in animal models of stress and in human patients with depression. Furthermore, some miRNAs have been postulated as potential biomarkers of stress/depression (extensively reviewed by Dwivedi and Brites and Fernandes [124, 125]). miRNAs also may play important roles in the mechanism of action of antidepressants: for example, in early-life stress models, the downregulation of miR-451 was reversed after antidepressant treatment [126].

Regarding astrocytes, although the information available about the differential cargo of astrocyte-derived exosomes after stressful conditions is scarce, it is worth pointing out that several miRNAs that are up or downregulated in stress conditions are contained in exosomes secreted by astrocytes. These miRNAs have also been described to play a role in the neurogenic process (Tables 2 and 3). Interestingly, miRNAs contained in astrocyte-derived exosomes are differentially enriched as compared to their levels in astrocytes [127], suggestive of their unique role in cellular communication. Moreover, many of the miRNAs contained in astrocytes can be modulated by different stimuli (see Table 4). All these data lead us to postulate astrocyte-derived exosomes as potential modulators of proliferation, migration, and/or differentiation of NSPCs within the neurogenic niche, and that changes in exosomal release as well as in their miRNA cargo can play a role in neurogenesis under stress conditions, in a similar fashion as it has been described for other CNS pathologies.

## 9. Conclusions and Future Perspectives

**Figure 1:**
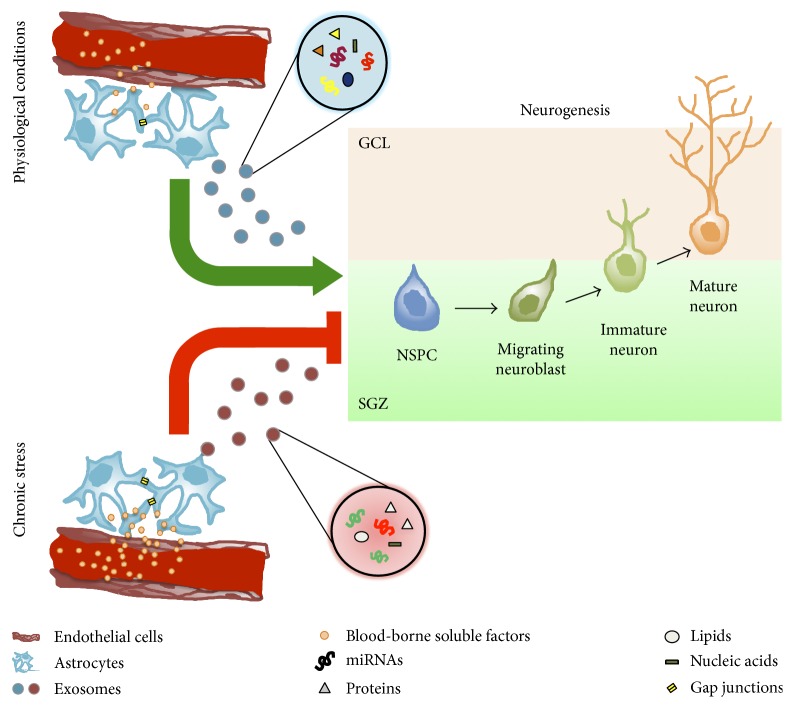
Blood-borne soluble factors reach astrocytes in the neurogenic niche, thus triggering the release of exosomes. In physiological conditions, the content of their cargo may exert a positive modulatory effect over one or more neurogenic stages (e.g., enhancing proliferation, and differentiation). During pathological conditions such as chronic stress, astrocytes respond to blood-borne soluble factors (e.g., corticosteroids and cytokines) by releasing exosomes with a cargo that may have a negative modulatory influence over one or more neurogenic stages. Astrocytes may in turn communicate with each other through gap junctions and/or by exosomal release. This may partly explain the decrease in differentiation and proliferation observed under such conditions. Note that the exosomal content under pathological or physiological conditions may differ in terms of the identity of the molecules (e.g., different types of miRNAs or proteins) and/or in their overall quantity. GCL: granule cell layer; SGZ: subgranular zone; NPSC: neural stem/precursor cell.

The production and proliferation of neural lineages (neurons, astrocytes, and oligodendrocytes) are a complex phenomenon tightly regulated by a multiplicity of factors. This regulation is susceptible to profound modifications when the homeostasis of the environment changes due to acute or chronic disorders. In the case of chronic stress, the observed modifications in the neurogenic niche (i.e., a decrease in NSPC proliferation/differentiation) lack a solid molecular explanation. Astrocytes may be key players to further understand on how and why the neurogenic niche responds the way it does in physiological and pathophysiological conditions. This is especially true in the case of the SGZ, where, due to their proximity with the vasculature, astrocytes may respond to factors in circulation (e.g., corticosteroids) to influence the behavior of the neurogenic niche [18]. We propose that a putative mechanism by which astrocytes exert their influence is through exosomal delivery of specific miRNAs. This could provide a finely tuned regulatory system, acting through two mechanisms: the first one is related to the unique membrane protein footprint that would enable astrocyte-derived exosomes to target specifically some, but not all, cell types of the neurogenic niche, and the second one is related with the miRNA cargo that most probably is unique under certain conditions. This could provide an exquisite temporal and spatial regulation for every single cell type implicated during the whole process of neurogenesis (Figure 1).

## Figures and Tables

**Table 1:** Effect of stress over neurogenesis mediated by astrocytes.

Type of stress	Type of study	Cellular effect	Molecular mechanism	References
Acute and chronic induced by dexamethasone	*In vivo*	Growth inhibition of astrocytes	After inducing cell cycle exit by reduction of cyclin D1 and increase of p27	[128]
	*In vitro*	Inhibition of NSPC proliferation (cultured with CM of stressed astrocytes)	By altered expression of neurotrophic factors (*BDNF*, *NGF*) and mitogenic factors (*BFGF*, *VEGF*) and death-inducing factors (FasL, Trail, Tweak, and TNF*α*)	
Acute induced by dexamethasone or corticosterone	*In vitro*	Inhibition of astrocytes proliferation	By inducing reduction of GR expression	[129]
Chronic induced by administration of ACTH	*In vivo*	Inhibition of astrocytes proliferation	By inducing reduction of GR expression	
Acute and chronic	*In vivo*	Regulation of mRNAs in a cell type-dependent fashion	By glucocorticoids receptors	[130]
Acute	*In vivo*	Increase hippocampus cellular proliferation	Increase of astrocytes FGF2 expression	[131]

**Table 2:** miRNA associated with neurogenesis present in astrocytes- and astrocytes-derived exosomes.

miRNA	Expression level	Cellular process	Molecular target	References
miR-9	Overexpression	Reduces axonal branching and neurite outgrowth	MAP1b	[132]
miR-9	Upregulation/overexpression	Promotes neuronal differentiation	Notch signaling, several targets	[133]
miR-9	Upregulation/overexpression	Promotes neuronal differentiation and dendritic branching, inhibits migration	TLX, REST, Rap2a, and stathmin	[134]
miR-9	Upregulation/overexpression	Suppresses astrogliogenesis	Lifr-beta, Il6st (gp130), and Jak1 (jack/stat pathway)	[135]
miR-9	Upregulation/overexpression	Promotes neuronal differentiation and migration	TLX/Nre1, Foxg1, REST/NRSF, CoREST, Meis2, Gsh2, Islet1, Id4, and stathmin	[136]
miR-9	Overexpression	Mediates neural differentiation of ES cell	STAT3	[137]
miR-9	Overexpression	Promotes neuronal differentiation	Foxg1, Gsh2, SIRT1, and REST/NRSF	[138]
miR-9	Overexpression	Inhibits NSPC proliferation and facilitates NSPC differentiation	TLX	[118]
miR-9	Overexpression	Inhibits NSPC proliferation and facilitates NSPC differentiation	Hes1 (notch signaling)	[139]
miR-26a	Upregulation	Inhibits spine enlargement	RSK3	[140]
miR-26a	Downregulation	Prevents axonal regeneration	GSK3*β*	[141]
miR-26b	Upregulation	Promotes neuronal differentiation	Ctdsp2	[142]
miR-29a	Upregulation	Increase axonal branching	DCX	[143]
miR-34a	Upregulation	Promotes neural differentiation and synaptogenesis	TAp73, synaptotagmin-1, and sintaxin-1A	[144]
miR-34a	Upregulation	Inhibits neuronal differentiation, promotes proliferation	Numbl, NeuroD1, and Mash1	[134]
miR-34a	Upregulation	Promotes apoptosis, inhibits cell cycle progression and synaptic development	BCL-2, Cdk-4 Cyclin D2 synaptotagmin syntaxin-1A	[134]
miR-34a	Upregulation	Negatively regulate neurite outgrowth and dendritic branching		[134]
miR-125b	Upregulation	Promotes neuronal differentiation	BMP/TGF*β* signaling	[133]
miR-125b	Upregulation	Promotes neuronal differentiation	Nestin	[145]
miR-125b	Upregulation	Inhibits NSPC proliferation and promotes differentiation	Musashi1	[146]
miR-129	Upregulation	Determination of the bipolar cell identity in retina	Xotx2, Xvsv1	[147]
miR-135b	Upregulation/Overexpression	Promotes neuronal induction	BMP/TGF*β* signaling	[148]
miR-145	Upregulation	Promotes neuronal differentiation	OCT4, SOX2, and KLF4	[149]
miR-145	Upregulation	Promotes neuronal differentiation	SOX2, Lin28/let7	[150]
miR-221	Downregulated	Neurite guidance		[151]
Let-7 family	Upregulation	Pluripotency inhibitor promoting neural lineage, promotes neuronal differentiation	Lin28	[133]
Let-7 family	Upregulation	Promotes NSPCs differentiation	c-Myc, Lin28	[136]
Let-7b	Upregulation	Inhibits proliferation and promotes the differentiation of NSPCs	TLX, Cyclin D1	[152]
miR-543	Upregulation	Promotes neural stem cell differentiation and neuronal migration	N-Cadherin, TrappC8	[153]

**Table 3:** miRNA associated with neurogenesis enriched in astrocytes derived exosomes.

miRNA	Expression level	Cellular process	Molecular target	Reference
miR-25b	Overexpression	Promotes proliferation and differentiation of NSPCs	IGF signaling	[154]
miR-17-92	Overexpression	Increase axonal outgrowth	PTEN	[123]
miR-92a	Upregulation/overexpression	Inhibits the transition from radial glial cells to intermediate progenitors	Tbr2	[155]
miR-184	Upregulation	Inhibits differentiation and promotes proliferation of NSPCs	Numbl	[156]
miR-302	Upregulation	Block neural progenitor induction	BMP/TGF*β*, NR2F2	[157]
miR-96	Upregulation	Block neural progenitor induction	PAX6	[158]

**Table 4:** miRNA associated with neurogenesis modified after different stimulus.

miRNA	Expression level	Cellular process	Molecular target	Reference
miR-181a	Upregulated by morphine	Promote astrocyte-preferential differentiation of NSPCs	Prox1/Notch2	[159]
miR-23b	Upregulated by morphine	Adult neurogenesis	Morphine receptor expression (MOR1)	[160]
miR-190	Downregulated by fentanyl	Adult neurogenesis	NeuroD	[161]
miR-143	Upregulation by IGF-1	Promotes proliferation, neural differentiation, and cell survival	PDGFRA, PRKCE, MAPK7, DSSP, DMP-1, KRAS, and BCL-2	[162]
miR-181c	Upregulation by IGF1/LIF	Enhanced self-renewal of NSPCs	PTPN11, PTPN22, PTEN, Dusp6, PBX3, ZEB2, and IRF8	[162]
